# A subspace approach to blind coil sensitivity estimation in parallel MRI

**DOI:** 10.1186/1532-429X-16-S1-W1

**Published:** 2014-01-16

**Authors:** Derya Gol Gungor, Rizwan Ahmad, Lee C Potter

**Affiliations:** 1 Department of Electrical & Computer Engineering, The Ohio State University, Columbus, Ohio, USA; 2 Davis Heart & Lung Research Institute, The Ohio State University, Columbus, Ohio, USA

## Background

In parallel MRI, subsampled k-space data are simultaneously collected by multiple coils. Each coil introduces a sensitivity map (CSM) that is multiplied pointwise with the single image to be reconstructed. In ESPIRiT [1], for each pixel location in each coil, an eigen-decomposition is applied to small matrices to obtain CSMs. However, this approach can be time-consuming for larger imaging problems. Here, we exploit smoothness of the coil sensitivities in the image domain to model them as small finite impulse response (FIR) filters in k-space as in PRUNO [2]. Since pointwise-multiplication in image domain corresponds to convolution in k-space, parallel MRI problem can be expressed as a blind image deconvolution problem; consequently, a subspace approach [3] can be used to estimate the k-space coefficients of the CSMs.

## Methods

If y_i_, x and h_i _represent fully sampled k-space data, true image, and k-space coefficients of the i^th ^CSM, then the problem can be written as y_i _= x*h_i _= Xh_i_. Further, the multichannel convolution can be written as Y = XH. Thus, if x has full rank, then the null-space of Y is equivalent to the null-space of H. As a result, the null-space (equivalently row-space) vectors used to reconstruct y_i _from subsampled data in PRUNO, can be used for the estimation of k-space coefficients of CSMs efficiently using the following optimization problem: h = argmax_h _||Vh||^2^+μ||Rh||^2^, where R represents a low-pass filter, and V involves convolution matrices of filters obtained from rowspace vectors. For validation, real-time, free-breathing 3-fold cine data were collected on a 3T Siemens scanner with matrix size 161 × 144 × 12 × 48. For CSM estimation, a 161 × 24 fully sampled k-space was obtained from 3 consecutive time frames. Among 432 singular values, the largest 70 were used as row-space vectors. A Gaussian function was selected for the low-pass R. The eigenvector associated with the largest eigenvalue of V^H^V+μ R^H^R was calculated to yield the 8 × 8 estimated k-space coefficients of the CSMs for μ = 5. Finally, the sensitivities were normalized with their square-root sum-of-squares (SoS) image.

## Results

**Figure 1:**
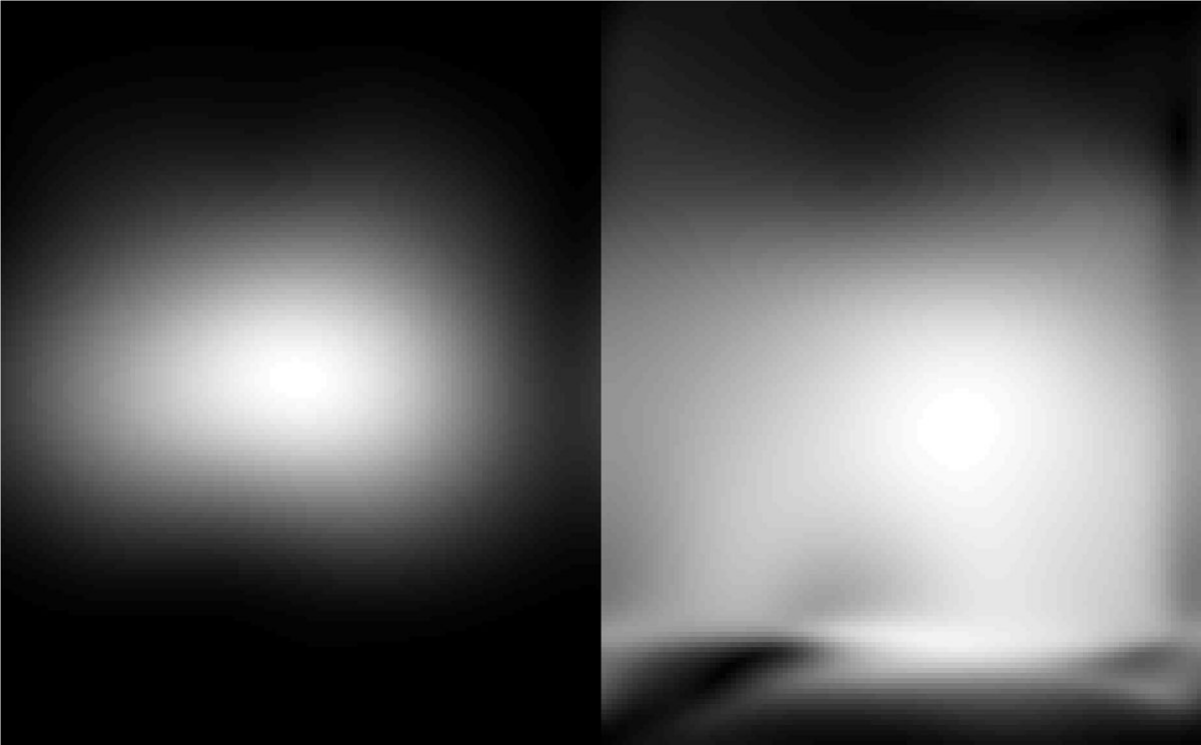
**Left: estimated coil sensitivity for one of 12 coils; Right: estimate normalized by the sum-of-squares of the estimated sensitivities**.

**Figure 2:**
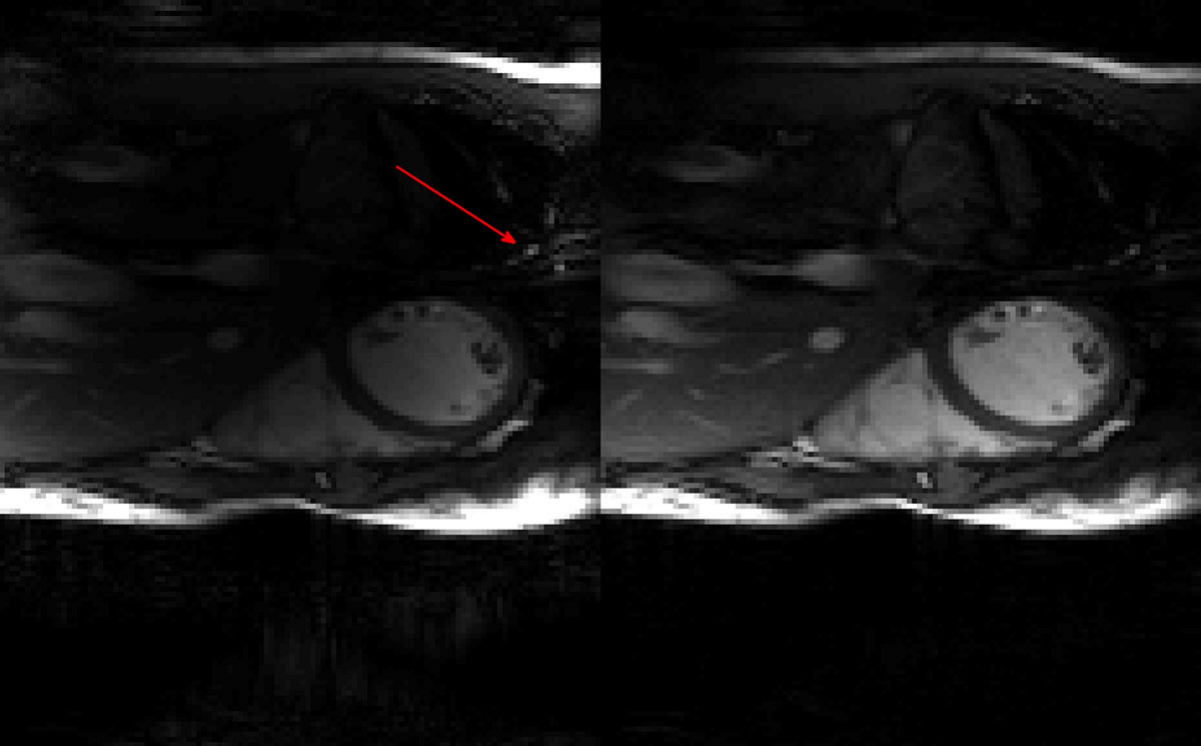
**SENSE reconstructions from the estimated sensitivities on the left and their SoS normalized versions on the right**. Red arrow points to a strong artifact.

Estimated CSM for one coil and its SoS-normalized version are demonstrated in Figure 1. SENSE[4] reconstructions for one of the frames are given in Figure 2 for the estimated CSMs and their SoS-normalized versions. As seen, inhomogeneity and artifacts existing in SENSE reconstruction is significantly reduced with the normalized CSMs. Compared to the image domain processing, the proposed k-space estimation of CSM was 10 times faster.

## Conclusions

The proposed k-space approach for CSM estimation using subspace methods and a simple normalization provides both low computational complexity and the flexibility to incorporate both regularization and a low-dimensional parameterization of the smooth CSMs.

## Funding

This work was supported by DARPA/ONR grant N66001-10-1-4090.
